# A randomized controlled longitudinal naturalistic trial testing the effects of automatic self transcending meditation on heart rate variability in late life depression: study protocol

**DOI:** 10.1186/1472-6882-14-307

**Published:** 2014-08-19

**Authors:** Zareen Amtul, Amanda Arena, Hussein Hirjee, Zaineb U Khan, Pramudith M Maldeniya, Ronnie I Newman, Amer M Burhan, Stephen Wetmore, Akshya Vasudev

**Affiliations:** Division of Geriatric Psychiatry, Department of Psychiatry, Western University, London, N6A 5 W9 ON Canada; Schulich School of Medicine and Dentistry, Western University, London, N6A 5C1 ON Canada; Department of Research and Health Promotion, The Art of Living Foundation, San Francisco, CA USA; Department of Family Medicine, Western University, London Health Sciences Centre, London, N6A 5C1 ON Canada; Division of Clinical Pharmacology, Department of Medicine, Western University, London, N6A 1H4 ON Canada; A2-607, Victoria Hospital, London Health Sciences Centre, 800 Commissioners Road East, London, N6A 5 W9 ON Canada

**Keywords:** Cardiovascular autonomic testing, Automatic self transcending meditation, Late life depression, Heart rate variability

## Abstract

**Background:**

The prevalence and socioeconomic cost of late life depression (LLD) is on the rise, while the response rate to antidepressant trials remains poor. Various mind-body therapies are being embraced by patients as they are considered safe and potentially effective, yet little is known regarding the effectiveness of such therapies to improve LLD symptoms. Among the mind-body therapies currently in practice, the results of our pilot study have shown that a particular meditation technique called *Sahaj Samadhi* Meditation, which belongs to the category of meditation termed automatic self-transcending meditation (ASTM) may have some promise in improving cardiovascular autonomic disturbances associated with LLD as well as ameliorating symptoms of depression and anxiety.

**Methods/Design:**

Patients between the ages of 60 and 85 with LLD will be randomized either to ASTM plus treatment as usual (TAU) or TAU alone to assess changes in cardiovascular autonomic parameters**,** neuropsychological symptoms of depression and anxiety as well as quality of life. The instructional phase of the intervention consists of 4 consecutive days of meditation training, after which participants are encouraged to meditate twice daily for twenty minutes each time at home. The intervention also includes once weekly follow up sessions for the subsequent 11 weeks. The planned study has one and a half year recruitment period. Participants will be assessed at baseline and at 4, 8, 12 and 24 weeks post intervention.

**Discussion:**

This study should provide a unique data source from a randomized, controlled, longitudinal trial to investigate the effects of a form of ASTM on cardiovascular autonomic and neuropsychological health in LLD.

**Trial registration:**

Clinicaltrials.gov NCT02149810, date registered: 05/28/2014.

## Background

Major depressive disorder in the elderly (above 60 years of age), also known as late life depression (LLD), is a common disabling condition that is associated with a high mortality rate, caused by either suicide or cardiovascular events [1], thus compelling appropriate treatment.

Depression in general, and LLD in particular, is increasingly recognized as a dysfunction in multiple underlying biological processes with an increased prevalence of comorbid cardiovascular autonomic disturbances compared to age-matched controls [2, 3], as reported by us previously [4]. Other studies conducted on depression across the human life span have reported similar results [5, 6], suggesting there may be an etiological link between autonomic dysfunction and LLD. This potential relationship necessitates further evaluation as it could provide invaluable insight into understanding the neurobiology of LLD and new treatment strategies.

Evidence shows that in a naturalistic setting, the response rate to an antidepressant trial of adequate dose and duration is often inadequate and can be as low as 30-40% in LLD [7]. In addition, antidepressant use is often associated with a number of adverse events which lead to frequent discontinuation of such treatment [8]. Hence there remains the need for additional safe and effective interventions [2].

Recently, various forms of non-pharmacological therapies loosely defined as mind-body therapies such as biofeedback, energy healing, meditation, guided imagery, and yoga [2, 9] are being increasingly embraced by patients. Such therapies have negligible side effects, are easy to administer and display beneficial effects on quality of life as well as comorbid anxiety. Unlike antidepressants that classically alter neurotransmitter levels [3], mind-body therapies target multiple organ systems and offer an added advantage over anti-depressant treatment alone by ameliorating both depression and cardiovascular health when offered in combination with antidepressants therapy.

Various forms of meditation have been employed as mind-body treatments. Automatic self-transcending meditation (ASTM) is a category of meditation that helps quiet the mind and induce physiological and mental relaxation while the eyes are shut. It utilizes relaxed attention to a specific sound value (*mantra*) according to specific criteria, in order to draw attention inward. This permits the mind to experience a restful but alert state of consciousness [10, 11]. ASTM specifically improves autonomic dysfunction among elderly with and without cardiovascular disease and has a positive impact on depressive symptoms and stress [12]. In a randomized controlled trial of elderly retirement home residents comparing ASTM with two other meditative techniques and treatment as usual (TAU), ASTM produced significantly greater improvements in cognitive function, cardiovascular function and quality of life than all other treatment conditions [12, 13]. Studies of ASTM in adults with cardiovascular disease further demonstrated improvements in cardiovascular function including heart rate variability (HRV) [13–16], an easily measurable cardiovascular autonomic variable. A subsequent meta-analysis of all-cause mortality rates among hypertensive elderly who had participated in stress reduction interventions found that ASTM practitioners had a 30% lower cardiovascular mortality rate than four other meditative or relaxation interventions [17].

These studies suggest that ASTM may be particularly well suited to elderly populations. However, it has not yet been shown if the benefits offered by ASTM would translate to elderly depressed subjects. We found through a pilot study that ASTM led to an improvement in HRV and was associated with reductions in symptoms of both depression and anxiety. The data also showed that the ASTM intervention did not induce any form of distress in participants, adding to the utility of this practice as a therapeutic tool. However, given that this was a pilot study, further investigation is required. Therefore we aim to assess ASTM in a large-sample randomized, controlled, longitudinal trial to confirm its effectiveness. In this paper, we provide a description of the protocol, participants’ inclusion/exclusion criteria, as well as cardiovascular and neuropsychological assessments that will be employed during the study.

## Methods/Design

### Aims

The primary aim of this study is to examine whether or not *Sahaj Samadhi* Meditation, a form of ASTM leads to increased HRV in patients with LLD. Additional aims are to examine its impact on symptoms of depression and anxiety and to determine if it leads to improvements in quality of life.

### Study design

The study aims will be addressed via a single-centre, single-blind, longitudinal randomized controlled naturalistic trial.

### Recruitment

Participants will be recruited from primary, secondary, and tertiary care centers in London, Ontario. We expect to recruit an average of two participants per week in a staggered manner, over a one and a half-year period. A telephone line will be available for study contact, concerns, and queries. The study will be advertised at key areas around the city including various community centers and libraries, as well as via electronic, print and web media.

### Participants

Research participants will be 96 elderly men and women, who have mild to moderate major depressive disorder (MDD). Diagnosis of MDD will be confirmed through a *S*tructured *C*linical *I*nterview for *DSM-IV-TR* (SCID) *Axis I* disorder [18].

### Eligibility assessment

Potential participants will be screened as per inclusion and exclusion criteria at the Geriatric Mental Health Program at London Health Science Center, London, Ontario. The Program includes two geriatric psychiatrists, registered nurses, and social workers, and serves adults over 65 from London and Middlesex County [19]. The Program accepts referrals from family physicians and other healthcare providers. In order to meet the inclusion criteria, participants must be between 60 and 85 years of age, have a diagnosis of mild to moderate MDD with a 17 item Hamilton Depression Rating Scale (HAMD-17) score of 8 to 22 [20], be of good general physical health, have sufficient hearing to be able to follow verbal instructions, be able to sit without physical discomfort for 45 minutes and be able to attend 4 initial ASTM training sessions. They must also agree to home practice of ASTM, and to attend 75% of weekly follow-up sessions. If being treated with antidepressant medications, they should be at therapeutic doses for a minimum of four weeks. Exclusion criteria include participation in other similar studies, other significant mental health diagnoses (including dementia, substance dependence, post traumatic stress disorder, obsessive compulsive disorder, bipolar disorder, neurocognitive disorder and personality disorders), high risk of suicide as elicited by clinical interview, psychotic episodes within the past 12 months, head trauma within the past 6 months, use of tricyclic antidepressants, monoamine oxidase (MAO) inhibitors, serotonin noradrenaline reuptake inhibitors (SNRI) or antipsychotic agents, practice of any type of formal meditation, mindfulness or breathing techniques, severe cardiovascular disease (myocardial infarction, stroke or transient ischemic attack) in the past 12 months, or any history of neurological disease (including Parkinson’s disease), seizures, or diabetic neuropathy.

### Baseline assessments

Once written consent is obtained from a potential participant, a baseline assessment will be conducted to ascertain the participant’s eligibility. Upon selection, each participant will undergo a *structured clinical interview; SCID*. The neuropsychological assessment consists of four examiner-rated questionnaires including the Cumulative Illness Rating Scale for Geriatrics (CIRS-G) [21], Hamilton Depression Rating Scale (HAMD-17) [20], Physical Activity Scale for the Elderly (PASE) [22], and Mini Mental State Examination (MMSE [23]). Four self-rated questionnaires will also be used to obtain self-reports of depression, anxiety, quality of life, and antidepressant side effects. These include the Geriatric Depression Scale (GDS) [24], Geriatric Anxiety Inventory (GAI) [25], Quality of Life Profile: Seniors Version (QOLPSV) [26], and Toronto Side Effects Scale (TSES) [27], respectively.

#### Structured Clinical Interview for DSM-IV-TR Axis I Disorders (SCID)

The SCID-I is a semi-structured interview for diagnosing current and past DSM-IV Axis I disorders and has excellent face and internal validity for diagnosing major depression [18].

#### Hamilton Depression Rating Scale (HAMD)

The HAMD is a 17-item structured scale administered to individuals diagnosed with unipolar major depression. It has been devised to quantify the severity of the depressive symptoms of the patient, based on the necessary information elicited by the interviewer [20]. It has been used in innumerable clinical trials for monitoring the change in mood state over a period of time.

#### Physical Activity Scale for the Elderly (PASE)

The PASE is a ten-item questionnaire assessing the frequency and intensity of engagement in physical activities, for individuals aged 65 and older, including walking, housework, sports, and other recreational activities over a one-week period [22].

#### Mini Mental State Examination (MMSE)

The MMSE is a quick test for grading the cognitive function or decline. Consisting of 11 questions and requiring less than ten minutes to complete, it can be used to screen for mild cognitive impairment or dementia [23].

#### Geriatric Depression Scale (GDS)

The 15-item GDS is a shortened version of the original, 30-item GDS questionnaire used to rate depression in the elderly [24]. The 15-item version has been shown to be effective in screening for depression in cognitively intact older adults [28].

#### Geriatric Anxiety Inventory (GAI)

The GAI is a 20-item questionnaire used to detect the presence of anxiety symptoms in older adults with or without a comorbid diagnosis of Generalized Anxiety Disorder (GAD). It has been found to be psychometrically sound and effective at detecting either the presence of GAD (using a cut off point of 8/9) or any anxiety disorder (using a cut point of 10/11) [25].

#### Clinical Global Impression-Improvement (CGI-I)

The CGI provides a summary of changes in an individual’s clinical status from the previous assessment and uses a seven-step categorical scale from 1 (very much improved) to 7 (very much worse) [29].

#### Quality of Life Profile: Seniors Version (QOLPSV)

The QOLPSV (short) is a 54-item questionnaire assessing quality of life in older adults using a model which focuses on more than just the absence of disease. The questionnaire aims to measure the “degree to which a person enjoys the important possibilities of his/her life” in the domains of Being (covering physical, psychological, and spiritual wellness), Belonging (evaluating a person’s fit in their social and physical environment), and Becoming (including daily activities as well as personal goals and aspirations) [26].

#### Toronto Side Effects Scale (TSES)

The TSES is a comprehensive, 32-item score sheet assessing the frequency and severity of various side effects which may occur with antidepressant therapy. It provides information for the cost/benefit analysis of a treatment since antidepressant medications tend to vary based on their side-effect profiles more than their remission rates [27].

### Baseline demographic values

Baseline demographic values, which could act as covariates or confounds for the tested treatment modality, will be also collected. These include medication use (including over the counter medication), number of previous depressive episodes, age at onset of first depressive episode, body mass index (BMI), smoking behavior, alcohol and coffee intake.

### Medication adherence

During the study period, specific antidepressants including Selective Serotonin Reuptake Inhibitors (SSRIs), (such as, fluoxetine, fluvoxamine, paroxetine, citalopram, escitalopram, paroxetine), Noradrenergic and Specific Serotonergic Antidepressants (NaSSAs) (such as, mirtazapine), bupropion, and anxiolytics (such as, benzodiazepines, zopiclone and trazodone) will be allowed as they have been found to be safe on autonomic variables [30]. Dosages will be optimized for a period of at least 4 weeks prior to enrollment and dosage modifications will also be permitted and monitored by pill count and history at follow-up appointments.

### Randomization and blinding

After baseline assessments, participants will be randomized in blocks of four to ASTM + TAU or TAU equally (1:1) using an online random allocation software v2.0 [31]. Concealment of randomization will be ensured by an administrative staff member keeping the randomization list in a locked cabinet inaccessible to other research staff. Immediately after each randomization, the participants will be notified of treatment allocation. Block randomization will allow ASTM treatments to be conducted in groups of four participants, minimizing therapist time while offering the potential benefits of group therapy. Pre-randomized information will be stored using unique identifiers on a secure database. Outcome assessors will be blinded to treatment. It will not be possible to blind participants to intervention status.

### Interventions

#### ASTM treatment arm

Following the initial HRV and neuropsychological measurements, participants in the ASTM arm will undergo *Sahaj Samadhi* Meditation training in groups of four by certified Sahaj Samadhi Meditation teachers under the supervision of one of the study authors (RN). This involves participating in 120-minute sessions on each of four consecutive days. Participants will individually be given a mantra on day one, and then be instructed in use of the mantra according to specific criteria over the four session program in groups of four. The training will be done in a quiet room within the hospital. This will be followed by weekly 60-minute follow up sessions for the 11 subsequent weeks. In addition, participants will be asked to practice ASTM at home for 20 minutes twice daily over the study period (24 weeks) and to log practice frequency and other noteworthy observations in the log sheet provided to them.

#### TAU control arm

**Figure 1:**
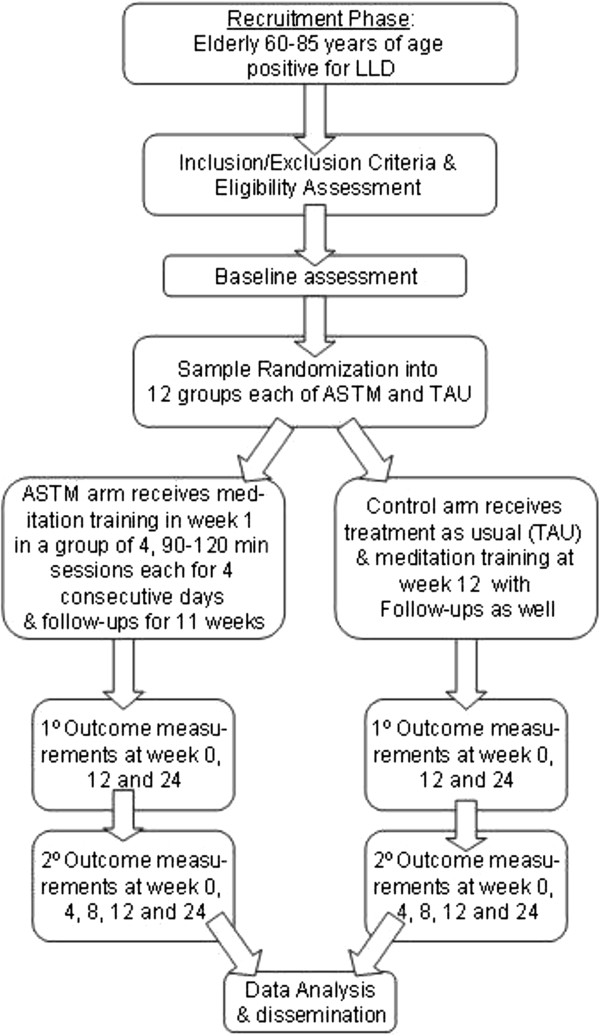
**Participant flow chart.**

The control group will continue to receive treatment as usual (TAU) including antidepressant medications and/or psychotherapy. After week 12, TAU arm participants will be offered the opportunity to learn ASTM and attend follow up meditation sessions. However, no assessments will be done or information collected on the TAU arm from week 12 onwards. Figure 1 depicts the participant flow chart.

### Outcome measurements

#### Primary outcomes

These involve cardiovascular autonomic assessments through electrocardiogram (ECG) and blood pressure measurements along with the measurement of respiratory excursions. These will be taken prior to the intervention (baseline or week 0), 12 and 24 weeks post-intervention for both ASTM and TAU groups. Participants from both arms will be asked to report to the Laboratory for Brain and Heart Health, Labatt Health Sciences Building, on the Western University campus. Heart rate will be monitored using an ECG with two adhesive leads or surface electrodes placed on the chest and one on the abdomen. Blood pressure will be measured using two small cuffs placed on a finger and wrist (Finometer) and an arm cuff device. The finger cuff continuous measures of blood pressure will be confirmed against arm cuff values obtained periodically by an automated sphygmomanometer (Dinamap). A respitrace bellows placed around the abdomen will provide information on respiratory excursions.

#### Secondary outcomes

Depression and comorbid anxiety symptoms will be assessed via the administered and self-rated scales (i.e. HAMD-17, Clinical Global Impression (CGI), GDS and GAI) at baseline (week 0) and 4, 8, 12 and 24 weeks post-intervention. Physical activity, antidepressant side effects and quality of life will also be assessed at the same time using the PASE, TSES and QOLPSV, respectively. Antidepressant medication adherence threshold will be set at 80% of pills consumed. Table 1 shows the primary and secondary outcome measures for all participants from weeks 0 to 24.

**Table 1:** **Primary and secondary outcome measures for all participants from weeks 0 to 24**

Evaluations	Enrol-ment	Randomisation/Interventions	Follow-ups			
	Week 0	Week 1	Week 4	Week 8	Week 12	Week 24
Assessment of eligibility	✓					
Informed Consent	✓					
Heart rate variability, Blood pressure, Respiratory excursion	✓				✓	✓
***To be completed by examiner:***	✓					
SCID						
Suicide risk assessment	✓		✓	✓	✓	✓
Concomitant medication adherence record	✓		✓	✓	✓	✓
CIRS-G	✓					
HAMD	✓		✓	✓	✓	✓
PASE	✓		✓	✓	✓	✓
CGI			✓	✓	✓	✓
MMSE	✓					
***To be completed by participant:***	✓		✓	✓	✓	✓
GDS						
GAI	✓		✓	✓	✓	✓
QOLPSV	✓		✓	✓	✓	✓
TSES	✓		✓	✓	✓	✓

List of abbreviations:

*HAMD-17*: 17 item Hamilton Depression Rating Scale, *CGI*: Clinical Global Impression, *GDS*: Geriatric Depression Scale, *GAI*: Geriatric Anxiety Inventory, *MMSE*: Mini Mental State Examination, *PASE*: Physical Activity Scale for the Elderly, *CIRS-G*: Clinician Illness Rating Scale-Geriatric version, *QOLPSV*: Quality of Life Profile: Seniors Version.

#### Patient adherence to the treatment

The research staff will record the number and duration of all attended ASTM sessions, compliance to home practice and the number of missed sessions. Based on this information, we will calculate the proportion of missed sessions as a quantitative index of treatment adherence.

### Statistical analysis

The design for this investigation is a split-plot partial hierarchical factorial design analysis of variance with two primary treatment conditions (ASTM + TAU vs TAU) tested at fixed time intervals.

#### Sample size

The primary focus of this study is the interaction effect of treatment on HRV. On the assumption that this effect size is medium (Cohen’s f = 0.25), calculations (using G*Power) [32] indicate that a sample size of 80 (4 in each of 20 groups) yield power estimates of 0.99 for both the interaction and the main effect of time. We intend to test a total of 96 participants to cover a conservative estimate of 20% loss to follow up. Based on the data obtained from the pilot study we consider these figures to be feasible.

#### Treatment effect

The power estimate for relevant simple main effects of time for the ASTM treatment condition is 0.93 (or 0.83 Bonferroni once adjusted). Simple main effects of treatment at each level of time are not expected for early time periods, but could be present at 24 weeks with a significant linear by linear interaction of time and treatment. This contrast is estimated to have a power of 0.72 assuming a medium effect, but the effect might well be stronger with the cumulative effects of time.

#### Primary outcomes

An upward deflection in an ECG is referred to as R wave and R-R is the interval between successive Rs. The HRV will be calculated by the standard deviation of all normal R-R intervals (SDNN) on the ECG, the root-mean square of successive differences (RMSSD), as well as the number of R-R intervals differing by >50 milliseconds from adjacent intervals (NN50) according to time-domain analysis that are based on beat-to-beat or NN intervals. Time-domain analysis will be preferred over spectral density analysis as it treats the NN interval sequence as an unordered pairs of intervals [33], whereas spectral density analysis presents information on the power distribution (variance) of the ordered NN intervals across frequencies [34].

#### Secondary outcomes

All secondary variables measured at the five time points will be analyzed with a similar design (i.e. a 2×5 split plot factorial design analysis of variance with groups crossed with time but nested in treatment intervention). With the total sample size of 80, this yields power estimates of 0.999 for both time and the treatment x time interaction.

### Fidelity/confidentiality

All outcome data will be stored on a encrypted database located on a secure server housed at the London Health Sciences Centre.

### Participant retention

Effort will be made to promote participant retention by allowing flexibility of screening and follow-up assessment times, offering the meditation course at no cost, and offering monetary compensation for participant time.

### Training of research staff

Research staff will receive training in the administration of relevant scales by AV. The CIRS-G will be conducted by one of the psychiatrists on the study team. All research staff will also be trained on the SCID [18] using the proprietary manual and instructional videos.

### Ethics and safety

Ethical approval has been obtained from the University of Western Ontario Health Sciences Research Ethics Board (UWO HSREB#103966).

### Risk screening

At both the baseline assessment and during frequent follow-up interviews, any concerns of acute distress and active suicidality/self-harm will be assessed by the psychiatrists on the team. Individual patients will be withdrawn from the study if there is reason to believe that continuation with the program would be deleterious for their mental health or safety. Particularly, participant involvement will be terminated if there are notable increases in the total HAMD score and/or suicidal ideation. In case of imminent risk, the patients will be referred to the Centralized Emergency Psychiatry Service at London Health Sciences Centre. All scientific instruments and the therapeutic modality have been previously shown to cause minimal to no discomfort. All sites have access to first aid and cardiopulmonary resuscitation in the event of an emergency. Participants will also be asked to indicate any discomfort, at which time the appropriate adjustments will be made, including discontinuation of the experiment for that participant if necessary. While participants are practicing ASTM techniques at their own homes, there are no additional perceived risks to their physical or mental health. As per the inclusion criteria, only patients at low risk for suicide will be recruited into the study.

## Discussion

This paper describes the protocol of an innovative and, to the best of our knowledge, the first randomized controlled longitudinal trial that aims to examine the efficacy of a non-pharmacological mind-body intervention (ASTM) to improve cardiovascular autonomic function, quality of life, and symptoms of depression and anxiety in an older adult population suffering from depression. This study has now received all necessary approvals. Recruitment has commenced and it is aimed to be completed by December 2015. Methods have been put in place to ensure participants recruitment as per protocol. An extensive recruitment strategy has been devised to engage primary care practitioners and general public through radio talk shows, web, print and electronic advertisements.

Autonomic disturbance has been reported in patients with late life depression [2, 3]. The primary outcome measure assessing cardiovascular health (HRV) is a well-established autonomic measure that has been used in other trials [13–16]. It can be conveniently and quickly collected. To collect the primary outcome measures collaboration has been set up with a neurovascular research laboratory at Western University.

The secondary outcome measures (HAMD, PASE, GDS, GAI, QOLPS, and TSES) will yield information on expected improvements in depression, physical activity, anxiety and quality of life. We shall be able to monitor any side effects due to antidepressant use and to feed this back to the participants for further discussion with their treatment provider, if needed.

There is currently limited evidence regarding the effectiveness of mind-body therapies in the elderly population. We have designed a robust randomized controlled trial which would allow us to confirm if one such mind-body therapy, ASTM improves cardiovascular autonomic imbalance and improves severity of depression and quality of life.

Heart rate variability can be seen as a manifestation of the interplay of the central nervous system and the autonomic nervous system on a beat-by-beat basis [35]. If there are positive effects of ASTM on HRV, then this form of meditation could potentially improve cardiovascular outcomes including myocardial infarction as well as mortality in patients with LLD. Patients with depression at the tested age range (60-85 years) usually develop a number of comorbidities, such as hypertension and diabetes mellitus, and often have a history of cardiovascular and cerebrovascular events for which they require appropriate medications (e.g. beta blockers, calcium channel blockers, and antiarrhythmics) which could influence the autonomic variables being measured. Hence, it would be impossible to completely exclude patients either with a history of cardiovascular episode or currently on cardiovascular medications that might affect the primary outcomes of our study. However, the randomization procedure will enable us to control these variables by placing the participants in one of the two treatment arms (ASTM + TAU and TAU alone) with equal likelihood, which will also make the findings of this naturalistic study more applicable to the population seen in the clinic routinely.

This study is a practitioner-administered program. Such programs tend to have higher rates of adherence and engagement than self-practice programs [36]. Based on the evidence from previous studies conducted by one of the co-investigators (RN), we have found that older adults without depression have a very low attrition rate when subject to ASTM [12]. The authors expect that the combination of participant observation of improvements in their psychological and physical health, as well as the steps (described above) taken to make study assessments non-cumbersome will help to maintain the retention rate of participants.

The ASTM program is highly transferable to other practitioners through training and is easy to implement in routine clinical care. As much of the cost of such an intervention is associated with the development and initial testing, once evaluated it has the potential to be adapted and rolled out with ease at relatively little cost to a large number of elderly. We suspect that ASTM can make LLD treatment cost effective by reducing both direct (i.e. hospitalization, physician visits, and medication) and indirect medical costs (including home care by informal caregivers and loss of productivity by the caregiver). This would be consistent with previous findings that ASTM reduces medical care utilization [37] and costs [38]. Although, there are no known direct risks of ASTM, some reported adverse effects include relaxation-induced anxiety, boredom, feeling addicted to the technique, and mild dissociation. However, such effects have been found mostly in individuals who practice meditative techniques for several years; no other short-term adverse events have been reported. Primary and secondary outcomes that will be measured via minimally invasive tests have also shown no direct risks. Prolonged use of finger and arm cuffs may cause numbness and discoloration, which resolve upon removal of the cuffs. ECG testing may cause some discomfort when electrodes are removed and the adhesive may cause some redness on the skin that would dissipate quickly at the end of the protocol.

## Conclusion

It is proposed that ASTM augmentation is an effective intervention that may ameliorate the autonomic disturbance associated with LLD, reduce symptoms of depression and anxiety, and improve quality of life compared to TAU.
